# SOCS1 regulates senescence and ferroptosis by modulating the expression of p53 target genes

**DOI:** 10.18632/aging.101306

**Published:** 2017-10-28

**Authors:** Emmanuelle Saint-Germain, Lian Mignacca, Mathieu Vernier, Diwakar Bobbala, Subburaj Ilangumaran, Gerardo Ferbeyre

**Affiliations:** ^1^ Département de Biochimie et Médecine Moléculaire; Université de Montréal, Montréal, Québec, H3C 3J7; Canada; ^2^ Department of Biochemistry, Medicine & Oncology, Faculty of Medicine, McGill University, Goodman Cancer Research Centre, Montreal, Quebec, H3A 1A3, Canada; ^3^ Immunology Division, Department of Pediatrics, Faculty of Medicine, University of Sherbrooke, Sherbrooke, Quebec, J1K 2R1, Canada

**Keywords:** suppressor of cytokine signaling, tumor suppressor, ferroptosis, senescence, KAP1

## Abstract

The mechanism by which p53 suppresses tumorigenesis remains poorly understood. In the context of aberrant activation of the JAK/STAT5 pathway, SOCS1 is required for p53 activation and the regulation of cellular senescence. In order to identify p53 target genes acting during the senescence response to oncogenic STAT5A, we characterized the transcriptome of STAT5A-expressing cells after SOCS1 inhibition. We identified a set of SOCS1-dependent p53 target genes that include several secreted proteins and genes regulating oxidative metabolism and ferroptosis. Exogenous SOCS1 was sufficient to regulate the expression of p53 target genes and sensitized cells to ferroptosis. This effect correlated with the ability of SOCS1 to reduce the expression of the cystine transporter SLC7A11 and the levels of glutathione. SOCS1 and SOCS1-dependent p53 target genes were induced during the senescence response to oncogenic STAT5A, RasV12 or the tumor suppressor PML. However, while SOCS1 sensitized cells to ferroptosis neither RasV12 nor STAT5A mimicked the effect. Intriguingly, PML turned cells highly resistant to ferroptosis. The results indicate different susceptibilities to ferroptosis in senescent cells depending on the trigger and suggest the possibility of killing senescent cells by inhibiting pathways that mediate ferroptosis resistance.

## INTRODUCTION

p53 is by far the most commonly mutated gene in human cancers with mutations present in 36% of all patients [1]. p53 acts mainly as a transcription factor suggesting that p53-target genes should have important functions in cancer biology. Combining chromatin immunoprecipitation and gene expression data of different cellular models revealed a number of p53 target genes that range between 122 to 3697 genes with little overlap between studies but including both genes that are activated or repressed by p53 [2,3,4,5]. A meta-analysis of different p53 and cell cycle regulatory networks revealed that p53 acts mainly as an activator through proximal promoter binding while gene repression is mostly indirect and dependent on the DREAM or RB/E2F complexes [5]. Identifying the key p53 targets that mediate tumor suppression and the cellular processes they regulate is a pressing goal for cancer research.

The tumor suppressor activity of p53 in mice does not correlate with the expression of p53 targets controlled by an acute DNA damage response [6]. For example, mice deficient for p21, Puma and Noxa, which are p53 target genes mediating cell cycle arrest and apoptosis, do not display the tumor prone phenotype typical of p53 null mice [7]. Genes controlling cell signalling, the cytoskeleton, DNA repair and ferroptosis were then identified as new candidates to mediate the tumor suppressor functions of p53 [4,6]. Genome wide analysis of p53 binding under conditions of acute or chronic stimulation revealed distinct p53 binding sites and regulated genes suggesting that upon chronic stimulation p53 DNA binding properties are regulated differently than in acute conditions [8]. The present state of knowledge indicates that context largely determines the transcriptional response to p53 activation. Hence, the identification of p53 targets in conditions where p53 regulates tumor suppression will help to identify important pathways and mechanisms to halt tumorigenesis.

Activation of p53 is often mediated by the DNA damage response that triggers a series of post-translational modifications on p53 preventing its degradation by E3 ubiquitin ligases such as MDM2 [9,10]. A similar mechanism is activated by the nucleolar protein p19ARF [11], the tumor suppressor PML [12] and several ribosomal proteins linking p53 activation to nucleolar stresses [13]. During cellular senescence induced by aberrant STAT5A stimulation both DNA damage and SOCS1 expression are required for p53 activation [14,15]. The specific role of SOCS1 in the senescence response to activated STAT5A and the p53 target genes modulated in this context remain for the most part unknown.

In order to identify and characterize p53 target genes whose regulation depends on SOCS1 we compared the transcriptome of cells that enter senescence in response to constitutive STAT5A-signaling with the transcriptome of cells that failed to do so due to inactivation of SOCS1. We define a set of SOCS1-dependent p53 target genes, some of which are downregulated in many human cancers. Interestingly, this set of genes included the ferroptosis regulators SLC7A11 and SAT1 as well as p53 target genes previously linked to the cellular response to oxidized phospholipids. Consistent with the gene expression analysis, overexpression of SOCS1 sensitized cells to ferroptosis. In contrast, induction of senescence by RasV12, STAT5A or PML did not sensitize cells to ferroptosis indicating different susceptibilities to ferroptosis depending on the senescence trigger. We also provide new insights into the mechanism of modulation of p53 by SOCS1 by showing that SOCS1 can stabilize p53 independently of its effects on serine 15-phosphorylation and that SOCS1 can form a complex with the p53 repressor KAP1.

## RESULTS

### SOCS1 inhibition affects a selective group of p53 target genes

**Figure 1:**
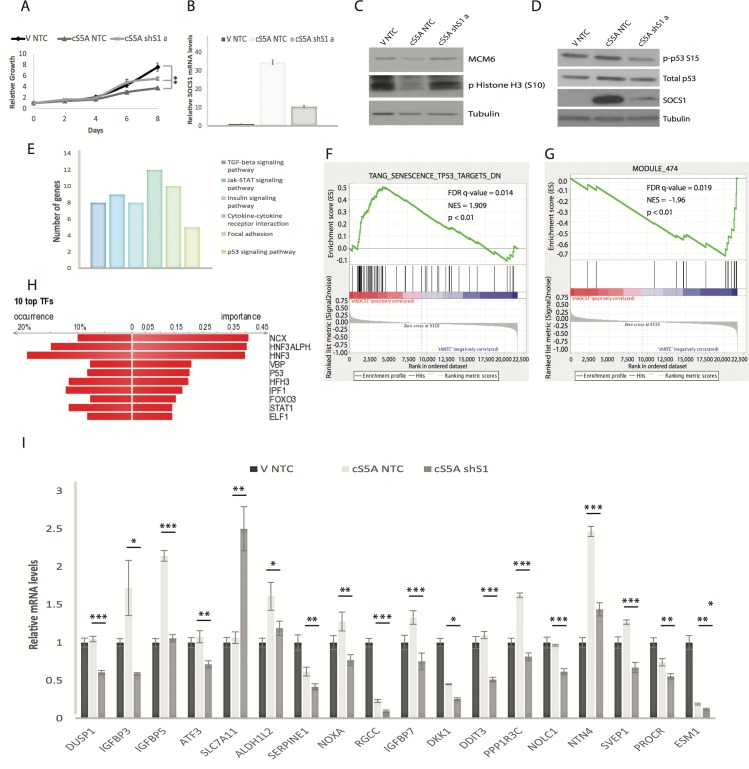
Microarray analysis identifies SOCS1-dependent p53 target genes (**A**) Growth curves. Normal human fibroblasts (IMR90) expressing viral oncoprotein E7 were retrovirally infected with either an empty vector (V) or with constitutively activated STAT5A (cS5A) and with either a control shRNA (shNTC) or a shRNA against SOCS1 (shS1 a). Cells were counted and plated for the growth assay. (**B**) SOCS1 mRNA levels were measured by qPCR using cells collected 7 days post infection, as in (A). (**C**) Western blots of IMR90 cells at day 7 post infection, as described in (A) for MCM6, phosphorylated Histone H3 (S10) and Tubulin. (**D**) Western blots of IMR90 cells described in (A) for p53, phosphorylated p53 at serine 15 (p-p53 S15) and SOCS1 levels. (**E**) DAVID analysis (Kegg pathway) of Affymetrix microarray experiment performed on triplicates of IMR90 cells expressing E7 and either constitutively active STAT5A (cS5A) combined with a control shRNA (NTC) versus cells expressing cS5A combined with an shRNA against SCOS1 (shS1), collected 7 days after infection. (**F**) Gene Set Enrichment Analysis (GSEA) of differentially regulated genes between the conditions in (D). (**G**) GSEA of differentially regulated genes. (**H**) DiRE analysis of genes differentially regulated between cS5A NTC and cS5A shS1 conditions of the Affymetrix microarray analysis. (**I**) QPCR validation in IMR90 cells expressing the same constructs as mentioned in (A), for the p53 target genes identified by the microarray analysis. All experiments were performed three times, error bars indicate SD of triplicates (growth curves) or standard errors of triplicates (QPCR), *= p<0.05, using the Student's t test. **=p<0.01, ***=p<0.005.

To investigate how SOCS1 modulates the p53 pathway we used a model of oncogene-induced senescence where p53 activation is dependent on SOCS1 [14,15]. In this model, a constitutively active allele of STAT5A (cS5A) is introduced into human fibroblasts IMR90-E7. These cells express the papillomavirus protein E7 to inactivate the retinoblastoma tumor suppressor, allowing us to focus on the p53 pathway contribution to cellular senescence. Expression of cS5A in these cells induced a cell cycle arrest and reduced the expression of proliferation markers (MCM6 and phospho-histone H3) that was rescued by expression of a small hairpin RNA against SOCS1 (Figure 1A-C). Consistent with the reported role of SOCS1 in p53 phosphorylation at serine 15, cS5A failed to induce this modification in cells expressing the shRNA against SOCS1 (Figure 1D). We then compared the transcriptome of IMR90-E7 cells expressing cS5A and co-expressing an shRNA against SOCS1 or a non-targeted shRNA using RNA purified from cells 7 days after introduction of cS5A. The microarray data was deposited in NCBI's Gene Expression Omnibus (GEO) GSE98216. Genes whose expression changed +/− 1.5 fold were used for pathway analysis with the online bioinformatics platform DAVID. The most significant pathways regulated by SOCS1 in cells expressing cS5A are indicated in Figure 1E and S1. They include genes coding for secreted proteins (Figure S1A-C) and a few genes in the p53-signaling pathway (Figure 1E and Table 1). The bypass of senescence induced by cS5A and the inhibition of p53 activity was also confirmed by a second shRNA against SOCS1 (shS1b), (Figure S2). Therefore, although these results confirm the requirement for SOCS1 in p53 phosphorylation and the senescence response to aberrant STAT5A activation, the pattern of p53 target genes identified does not include the classic p53 target genes associated to the DNA damage response.

**Table 1:** List of SOCS1-dependent p53 target genes identified by microarray analysis

Reported p53 target genes	Fold Change	PMID
DDIT3	−1.79	16917513
GADD45B	−1.74	23948959
IGFBP3	−1.85	20182617
PMAIP1	−1.65	19641509
SERPINE1	−1.52	17882266
LOXL1	−1.81	16888633
DKK1	−2.37	16888633
GDF15	−1.65	16888633
DDB2	−1.47	16888633
SLC7A11	1.58	25799988
ALDH1L2	−1.42	25799988
ABHD4	−1.43	25799988
BCAT1	−1.44	25799988
LRP1	−1.53	25799988
DUSP1	−1.52	25799988
PROCR	−1.52	25799988
RGCC	−2.49	17146433
IGFBP7	−1.72	21095038
SAT1	−1.56	27698118

Of note, the microarray data indicates many more known p53 target genes regulated by SOCS1 than found by DAVID. Relevant references for those genes are indicated by their PMID.

**Figure 2:**
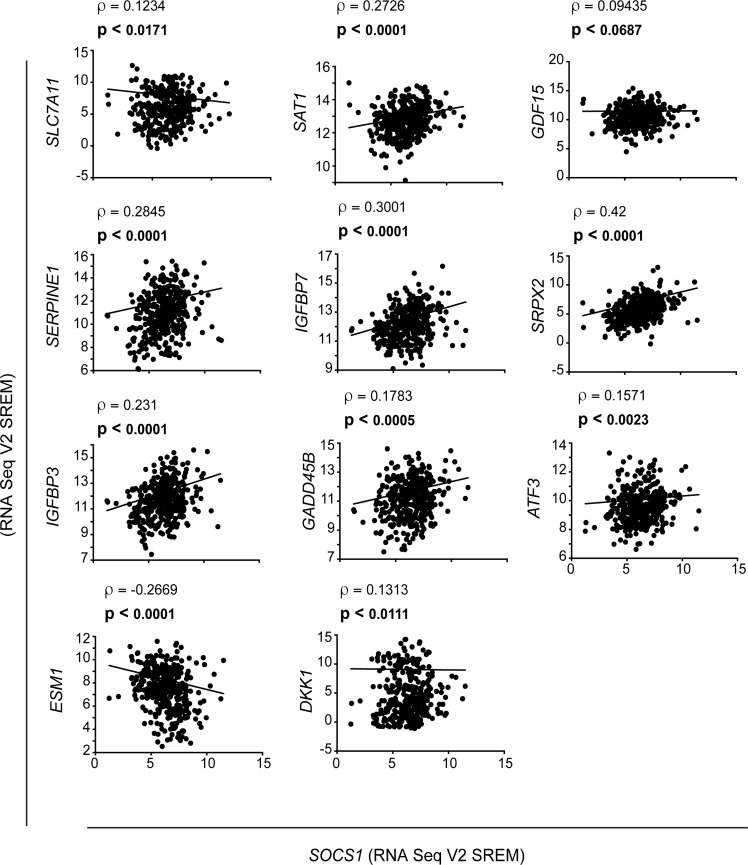
Correlation between SOCS1 and p53-target gene expression in hepatocellular carcinoma samples The TCGA dataset human HCC specimens was analysed to determine the correlation between the expression of *SOCS1* (*x*-axis) and the indicated p53 target genes (*y*-axis), as indicated by the slope. The Spearman correlation (ρ) and the *p* values are given at the top of each plot.

To further investigate whether SOCS1 modulates the p53 pathway, we performed Gene set enrichment analysis (GSEA) of the microarray data. We found that SOCS1-disabled cells have high levels of genes in the set TANG_SENESCENCE_TP53_TARGETS_DN that contain, genes upregulated by a dominant negative p53 in normal human fibroblasts (Figure 1F). This gene set mostly includes cell cycle regulated genes, which are known to be regulated by RB. However, the RB pathway was disabled in the IMR90-E7 cell line used in our experiments. These results thus imply a SOCS1- p53-dependent pathway that regulates cell cycle genes independently of the RB tumor suppressor. SOCS1-disabled cells also have a downregulation of the gene set MODULE_474 (http://robotics.stanford.edu/∼erans/cancer/) containing several genes upregulated in senescent cells such as IGFBP2, 3, 5, 6 and 7, NOV and SOCS3 (Figure 1G). Further proof that SOCS1 controls the expression of p53 target genes was obtained using the platform DiRE that analyses the promoters of gene sets for signatures of transcription factors. P53 was one of the top transcription factors associated to transcriptome changes induced by an shRNA against SOCS1 (Figure 1H). In addition, this algorithm identified 12 new candidates for p53-target genes (Table 2). Many of these genes are poorly expressed in human cancers suggesting that they could be novel tumor suppressors (Table 3). The microarray data was validated using qPCR for either known p53-target genes or the new candidates suggested by the DiRE platform (Figure 1I). We also used data in TCGA from hepatocellular carcinoma, a tumor type where promoter DNA methylation often silences SOCS1 expression [16]. We found a significant correlation between the expression of SOCS1 and the SOCS1-dependent p53 target genes defined in human fibroblasts (Figure 2). For several genes (SLC7A11, SAT1, SERPINE1, IGFBP7, GADD45 and ATF3) these correlations decreased in samples from p53 mutant tumors (Figure S3). Taken together, our results show that SOCS1 controls a unique set of p53 target genes.

**Table 2:** List of potential SOCS1 dependent p53 target genes identified by DiRE analysis

DiRE Analysis	Fold change	Function
ABCA8	−1.56	Transmembrane lipid transporter
ATF3	−1.69	Transcription factor, response to stress (16888633)
CITED4	−1.63	Transcriptional co-activator
CYGB	−1.51	Regulation of oxidative stress
DEPDC1B	1.62	Cell adhesion, mitosis regulation
ESM1	−1.53	Secreted factor, role in inflammation and cancer
KIAA1467	1.56	Uncharacterized protein
NOLC1	−1.87	Ribosome biosynthesis (21642980)
NTN4	−1.61	Role in metastasis (25590240)
PCDH10	−1.57	Cell adhesion and motility
PPP1R3C	−1.83	Regulation of glycogen metabolism
SRPX2	−1.83	Role in angiogenesis and migration
SVEP1	−1.67	Cell attachment
TMEM159	−1.73	Uncharacterized protein
ZNF2	−1.53	May be involved in transcriptional regulation

For previously linked p53 target genes, PMID is indicated in parenthesis as reference.

**Table 3:** Cancer vs. Normal expression of SOCS1-dependent p53 target genes identified by DiRE

	ABCA8		ATF3		CITED4		CYGB		DEPDC1B		ESM1		NOLC1		NTN4		PCDH10		PPP1R3C		SRPX2		SVEP1		TMEM159		SLC7A11	
Analysis types by cancer	Cancer vs. Normal																											
**Bladder Cancer**		4		3				1			3								1	1		1		2				
**Brain and CNS cancer**	1	1						4	2		4		1	1		2				1	6			1				
**Breast Cancer**		16		18				8	5		13		4	1		3			1	1	2	1		15	1		2	1
**Cervical Cancer**		2							1		1									4								
**Colorectal Cancer**		21		1	2		1		7		13		13			4				6	14	11		5		1	15	
**Esophageal Cancer**		2	1			2	2				1		2		1					5		2				1	1	
**Gastric Cancer**		5				2			3		5		1			3				1	2							
**Head and Neck Cancer**		10	1						1		1		1							9		2		2			2	
**Kidney Cancer**		4		2							4								5	1		2		4	2		4	
**Leukemia**			2	1		3	3			1	1			2			1						1			1		
**Liver Cancer**		3		1		1			2		1					2								1			1	
**Lung Cancer**		11		2					4							7				4	7	1		13			2	
**Lymphoma**			2	2			2		1				2										1			1		1
**Melanoma**		1											1											2				
**Myeloma**			1																									
**Other Cancer**		5	3	2			1		3		1					1	1				1	2	1	1			1	
**Ovarian Cancer**		6		1				1	1											1				2		1		1
**Pancreatic Cancer**		1					1		1												1				1	1	1	
**Prostate Cancer**			1	1		1						1				1		1		5			1					
**Sarcoma**		4		1							3								3		2	2		6				
		1	5	10		10	5	1																				
																												
					%																							

Red squares signal the number of studies showing upregulation and blue squares the number of studies showing downregulation. Cell color is determined by the best gene rank percentile for the analyses within the cell.

To investigate the biological importance of SOCS1-dependent p53 target genes we analysed their overlap with gene sets mediating specific p53-dependent responses. First, genes regulated by SOCS1 matched gene sets that regulate the response to chemotherapy (Figure S4A), which is largely influenced by the p53 pathway [17]. Second, SOCS1-disabled cells expressed high levels of genes upregulated by a dominant negative BRCA1 allele (Figure S4B), which also disrupts the p53 pathway [18]. Third, SOCS1-disabled cells have a decrease in the expression of genes that blocked angiogenesis in endothelial cells (Figure S4C). These genes include IGFBP3 [19] and COL4A2 which encodes the potent antiangiogenic factor canstatin [20]. It is well known that p53 inhibits angiogenesis [21,22], and our gene expression data suggest that SOCS1 modulates this p53 function as well. Finally, SOCS1-regulated genes also overlapped with a set of genes induced by oxidized phospholipids (Figure S4D), which has been recently linked to iron-dependent cell death or ferroptosis [23]. P53 sensitizes cells to ferroptosis by repressing the cystine transporter SLC7A11 [4] and inducing the polyamine metabolic enzyme SAT1 [24]. The regulation of those two genes in cS5A expressing cells was SOCS1-dependent (Table 1) suggesting a role for SOCS1 in ferroptosis.

The inhibition of SOCS1 expression in IMR90-E7 bypasses cS5A-induced senescence, raising the possibility that the defects in p53 target gene expression we described above are the consequence of senescence inhibition and are not directly linked to SOCS1. In IMR90 cells, where the retinoblastoma pathway remains intact, inactivation of SOCS1 does not bypass cS5A-induced senescence [14]. This is due to the known fact that the RB pathway is sufficient to regulate senescence in the absence of p53 [25,26]. We thus took advantage of this fact to investigate whether expression of p53 target genes still required SOCS1 in these cells.

**Figure 3:**
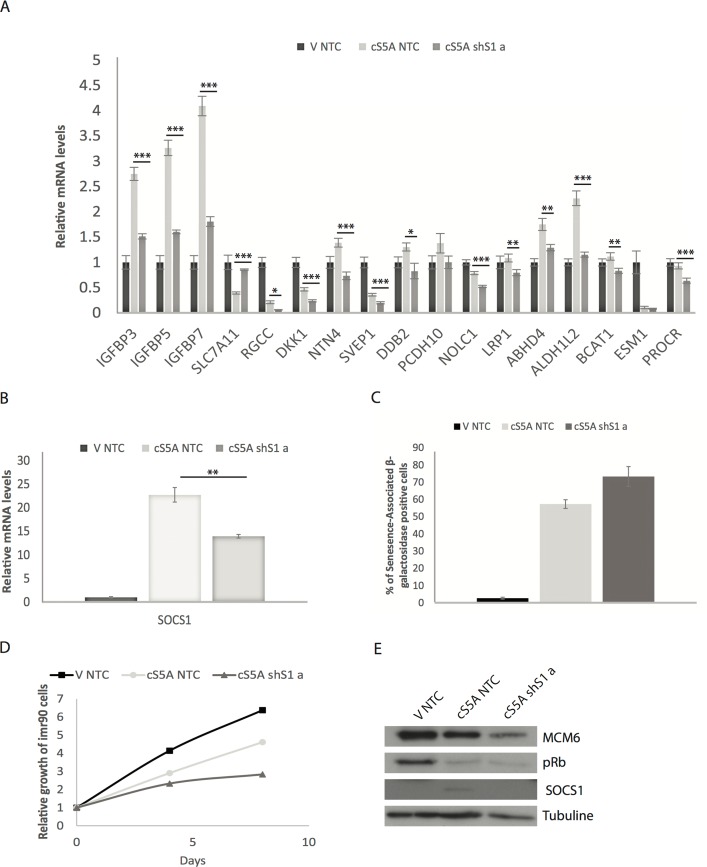
The regulation of p53 target genes by SOCS1 is not dependent on a disabled RB pathway (**A**) QPCR validation of the p53 target genes identified by microarray analysis but in normal IMR90 fibroblasts expressing either an empty vector (V) or a constitutively activated STAT5A (cS5A) and with either a control shRNA (shNTC) or an shRNA against SOCS1 (shS1). Cells were collected 7 days after infection. (**B**) SOCS1 knockdown efficiency measured by qPCR in the conditions described in (A). (**C**) Status of the cells was assessed at the day of RNA collection (day 7 post infection) with a Senescence-Associated β-Galactosidase staining. Positively stained and unstained cells were counted under a light microscope in order to obtain the percentage of senescent cells. (**D**) Growth curves. Normal human fibroblasts (IMR90) were retrovirally infected with either an empty vector (V) or with constitutively activated STAT5A (cS5A) and with either a control shRNA (shNTC) or a shRNA against SOCS1 (shS1 a). Cells were counted and plated for the growth assay. (**E**) Western blots of senescence markers (MCM6, pRb and SOCS1) of cells as in (A). Tubulin was used as a loading control. All experiments were performed three times, error bars indicate the standard errors of triplicates, * = p<0.05, using the Student's t test, **=p<0.01, ***=p<0.005

For most of the genes measured, disabling SOCS1 also inhibited the expression of p53 targets as well (Figure 3A-B) although cells remained senescent (Figure 3C-E). We conclude that the defects in p53 target gene expression we have seen after disabling SOCS1 are not the result of cell cycle re-entry after bypass of senescence and suggest a direct effect of SOCS1 on p53.

### SOCS1 is sufficient to activate p53 and regulate the expression of its target genes

**Figure 4:**
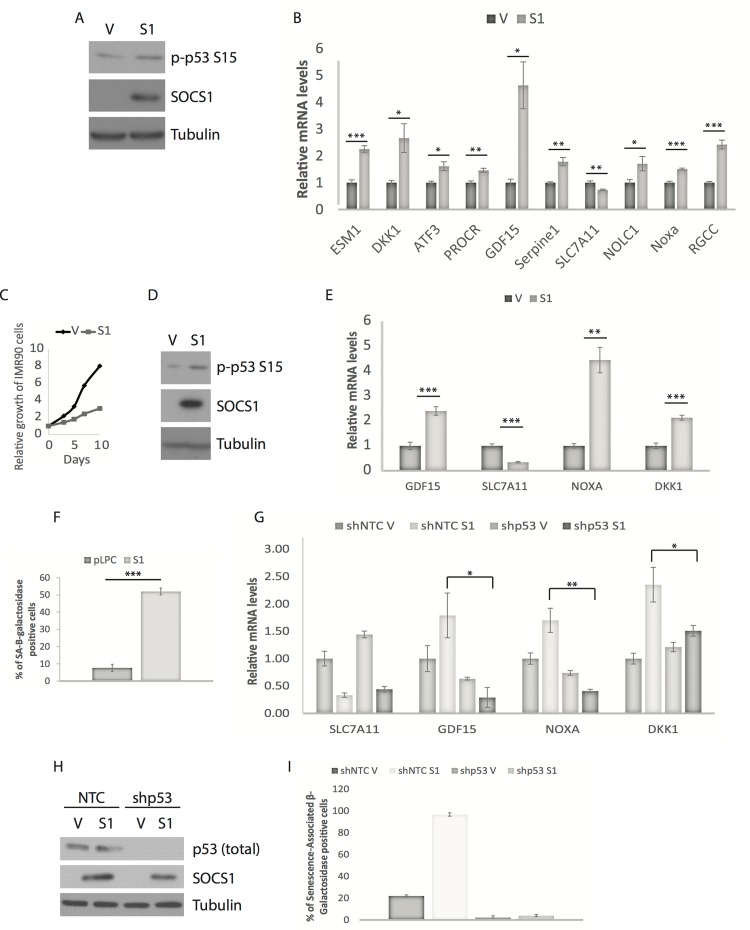
SOCS1 overexpression is sufficient to regulate the expression of SOCS1-dependent p53 target genes (**A**) Western blots of SOCS1 and phospho-p53 (p-p53 S15) in U2OS cells expressing either empty vector (V) or SOCS1 (S1). (**B**) QPCR for p53 target genes in cells as in (A). Cells were collected at day 5 or 7 post-infection. (**C**) Growth curves of IMR90 cells expressing either empty vector (V) or SOCS1 (S1). (**D**) Western blots of SOCS1 and phospho-p53 (p-p53 S15) in IMR90 cells expressing either empty vector (V) or SOCS1 (S1). (**E**) QPCR for p53 target genes in cells as in (C). Cells were collected at day-7 post infection. (**F**) Senescence associated β-galactosidase of IMR90 cells expressing either empty vector (V) or SOCS1 (S1). Cells were fixed and stained at day 12 post- infection. (**G**) QPCR of IMR90 cells expressing either a control shRNA (shNTC) or a shRNA against p53 (shp53) combined with SOCS1 (S1) or empty vector (V) to confirm that the genes in (E) are targets of p53. (**H**) Western blots for the indicated proteins in IMR90 cells expressing a control shRNA (NTC) or an shRNA against p53 (shp53) and also infected with a SOCS1 expressing vector (S1) or a vector control (V). (**I**) Senescence-Associated β-Galactosidase staining. Positively stained and unstained cells were counted under a light microscope in order to obtain the percentage of senescent cells. All experiments were performed three times, error bars indicate the standard errors of triplicates, * = p<0.05, using the Student's t test, **=p<0.01, ***=p<0.005.

Previous research established a function for SOCS1 as an adaptor protein facilitating the phosphorylation of p53 at serine 15 by the DNA damage response activated kinases ATM and ATR [14]. In culture, cells are exposed to high concentrations of oxygen and growth factors, increasing the probability of DNA damage by either replication stress or reactive oxygen species. We reasoned that enforcing SOCS1 expression might cooperate with these factors and engage the p53 pathway. Indeed, expressing SOCS1, in U2OS cells (Figure 4A-B) or IMR90 cells (Figure 4C-E) increased the expression of most of the genes that were positively regulated by p53 in cS5A-induced senescence and decreased the expression of SLC7A11, a gene that is repressed by p53. As reported before, SOCS1 was sufficient to trigger senescence in close to 50% of the cells overexpressing the protein (Figure 4F). The increase in expression of p53 target genes by SOCS1 was, for the most part, dependent on p53 since knockdown of p53 (Figure 4G-H) abolished their stimulation and prevented senescence (Figure 4I). However, the decrease in SLC7A11 was not blocked after knockdown of p53 suggesting that SOCS1 regulates the expression of this gene by additional mechanisms. Of note, not all p53 target genes that required SOCS1 in cS5A-induced senescence were induced by SOCS1 expression alone. The following genes (IGFBP3, GFBP5, DUSP1, ALDH1L12, IGFBP7, DDIT3, PPP1R3C, NOLC1, NTN4 and SVEP1) were not significantly induced by SOCS1 in either U2OS or IMR90 cells. In addition, RGCC, ESM1, ATF3 and PROCR were induced in U2OS but not in IMR90 cells. These results indicate that SOCS1 partially reproduces oncogenic STAT5A signaling to p53 but additional STAT5A functions also contribute to p53 activation.

### SOCS1 sensitizes cells to ferroptosis

**Figure 5:**
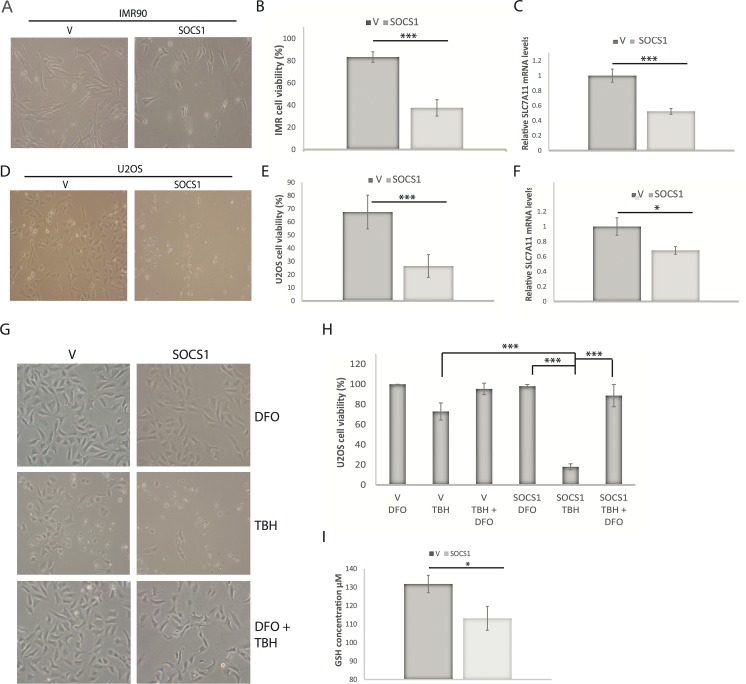
SOCS1 sensitizes cells to ferroptosis (**A**) Representative photos of IMR90 cells expressing an empty vector (V) or SOCS1 (S1) and treated 24 hours after plating with 88 μM tert-butyl-hydroperoxide (TBH). Cells were assayed for cell death 16 hours after treatment. (**B**) Quantification of cell viability portrayed in (A) by Trypan blue staining. (**C**) SLC7A11 mRNA levels measured by qPCR of IMR90 cells described in (A). (**D**) Representative photos of U2OS cancer cells expressing either V or S1 by retroviral infection and treated 24 hours after plating with 350 μM TBH for 16 hours. (**E**) Quantification of cell viability of U2OS cells as portrayed in (D) by Trypan blue staining. (**F**) Relative SLC7A11 mRNA expression measured by qPCR in U2OS cells expressing either V or S1 as described in (D). (**G**) Representative photos of U2OS cancer cells expressing either V or S1 by retroviral infection and treated 24 hours after plating with either 350 μM TBH alone, 100 μM Deferoxamine mesylate (DFO) alone or the combination of both drugs. (**H**) Quantification of cell viability portrayed in (G) by Trypan blue cell counts. (**I**) GSH quantification in U2OS cells expressing either V or S1. All experiments were performed three times, error bars indicate the standard deviation of triplicates, * = p<0.05, using the Student's t test, **=p<0.01, ***=p<0.005.

The mechanisms by which p53 prevents tumor progression include apoptosis, senescence and ferroptosis [27,28]. The latter is an iron-dependent cell death mechanism that involves reactive oxygen species and lipid oxidation and that does not depend on caspases or Bcl2 family members Bak and Bax [28]. The SOCS1-dependent p53 target genes SLC7A11 [4] and SAT1 [24] play key roles in ferroptosis suggesting that SOCS1 could be a novel regulator of this process. As anticipated, expression of SOCS1 in U2OS or IMR90 cells sensitized them to the ferroptosis inducer tert-butyl-hydroperoxide (TBH). This effect of SOCS1 correlated with its ability to reduce the expression of the cystine transporter SLC7A11 (Figure 5A-F) and was efficiently blocked by treatment with deferoxamine, an iron chelator known to inhibit ferroptosis (Figure 5G-H and Figure S4). Ferroptosis depends on the actions of oxidized lipids, which are detoxified by the glutathione (GSH)-dependent enzyme GPX4 [29]. Cystine is used in the cells to generate cysteine, a metabolite required for the synthesis of glutathione (GSH). As expected, expression of SOCS1 reduced the levels of GSH (Figure 5I) explaining in part its ability to sensitize cells to ferroptosis.

### The SOCS1-p53 axis is active in oncogene-induced senescence (OIS) but additional factors control ferroptosis in these cells

**Figure 6:**
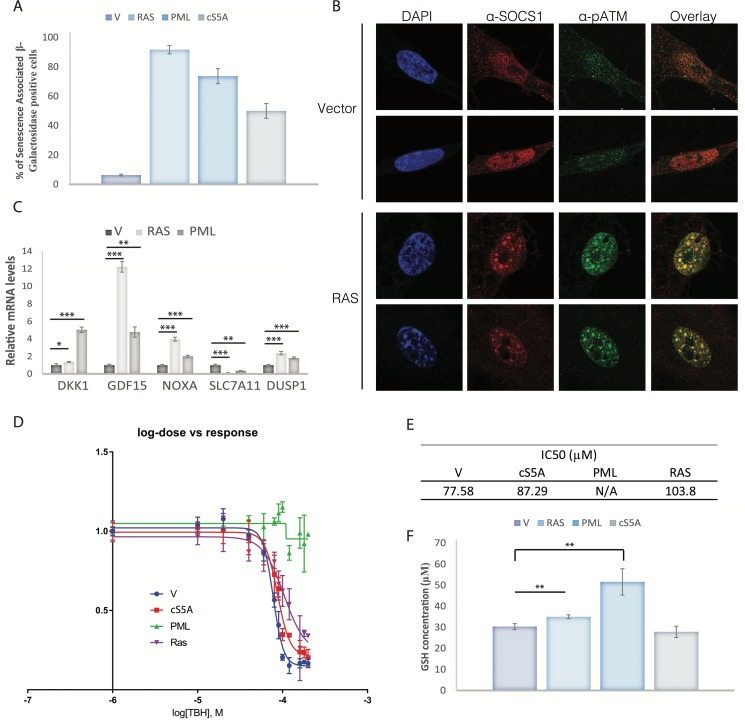
Ferroptosis sensitivity in senescent cells depends on the trigger (**A**) Senescence was assessed by staining cells for the Senescence-Associated β-Galactosidase in IMR90 cells expressing either a control vector (V), the RASV12 oncogene (RAS), PML or STAT5A (cS5A). (**B**) Immunofluorescence of SOCS1 (anti-SOCS1) and phosphorylated ATM at S1981 (anti-ATM) in IMR90 cells rendered senescent by overexpressing the RASV12 oncogene compared to IMR90 expressing a control vector (Vector). (**C**) QPCR for mRNA levels of SOCS1-dependent p53 target genes in IMR90 cells expressing a control vector (V) or rendered senescent by overexpression of RASV12 (RAS) or PML (PML). (**D**) IC50 curves of IMR90 cells overexpressing a control vector (V), the RASV12 oncogene (RAS), PML or STAT5A (cS5A). Cells were treated 24 hours after plating with 12 different doses (0, 10, 20, 40, 60, 80, 100, 120, 160, 180 and 200 μM) of tert-butyl-hydroperoxide (TBH). Cells were fixed and stained with Crystal Violet to assess cell death 16 hours after treatment. The dye was then solubilized with acetic acid 10% and measured with a spectrophotometer. (**E**) The value of IC50 of each condition graphed in D is presented. No IC50 could be calculated for PML as it was resistant at the doses used. (**F**) GSH quantification in IMR90 cells rendered senescent by overexpression of RASV12, PML IV or STAT5A, compared with empty vector control (V). All experiments were performed three times, error bars indicate the standard deviation of triplicates, * = p<0.05, using the Student's t test, **=p<0.01, ***=p<0.005.

OIS is triggered by activated oncogenes and involves replication stress, mitochondrial dysfunction, reactive oxygen species and the DNA damage response [30,31,32]. We anticipated that SOCS1 is generally upregulated during OIS and not only in the specific case of cS5A-induced senescence. This is because senescent cells secrete a variety of inflammatory mediators that activate JAK-STAT signaling [33], the major regulator of SOCS1 expression [14,34]. We thus induced senescence in IMR90 normal human fibroblasts with constitutively active RAS (RASV12), cS5A or the tumor suppressor PML, that acts downstream of RAS and cS5A to induce senescence (Figure 6A). As published before for cells expressing cS5A [14], we found SOCS1 localizing with phospho-ATM [35] in DNA damage foci in senescent cells, which are known to contain phosphorylated p53 at serine 15 [36] (Figure 6B). Also, induction of senescence by RASV12 or PML led to induction of SOCS1-dependent p53 target genes and the downregulation of the p53-repressible gene SLC7A11 (Figure 6C). Hence, despite the differences in the mechanisms triggering senescence by RasV12, STAT5A, SOCS1 or PML, they regulate a common set of p53 target genes.

Next, we performed ferroptosis assays in cells expressing RASV12, STAT5A or PMLIV using the ferroptosis inducer TBH and the ferroptosis inhibitor deferoxamine. For this assay we plated the same amount of cells for each condition and found that neither RasV12 nor STAT5A sensitized cells to ferroptosis, despite both having an increase in endogenous SOCS1 expression and a reduction in SLC7A11. Intriguingly, PML expression turned cells highly resistant to ferroptosis providing a mechanistic insight into a ferroptosis resistance pathway in senescent cells (Figure 6D-E). Glutathione levels correlated with ferroptosis resistance in senescent cells (Figure 6F) suggesting compensatory mechanisms of GSH biosynthesis as a possible mechanism for resistance to ferroptosis. Targeting anti-ferroptosis pathways activated in senescent cells might selectively kill them in the same way as targeting anti-apoptotic proteins.

### SOCS1 activates p53 via both phosphorylation and stabilization

The pattern of p53 target genes regulated by SOCS1, including ferroptosis regulators, is unique and anticipates unique mechanisms of p53 activation by SOCS1. So far, SOCS1 has been linked to the regulation of serine 15 phosphorylation of p53 [14,15], a modification that is also efficiently induced during an acute DNA damage response, which does not necessarily evolve into a permanent senescent cell cycle arrest [37,38]. Therefore, the ability of SOCS1 to induce senescence must involve additional mechanisms capable of changing the quality of the p53 response.

**Figure 7:**
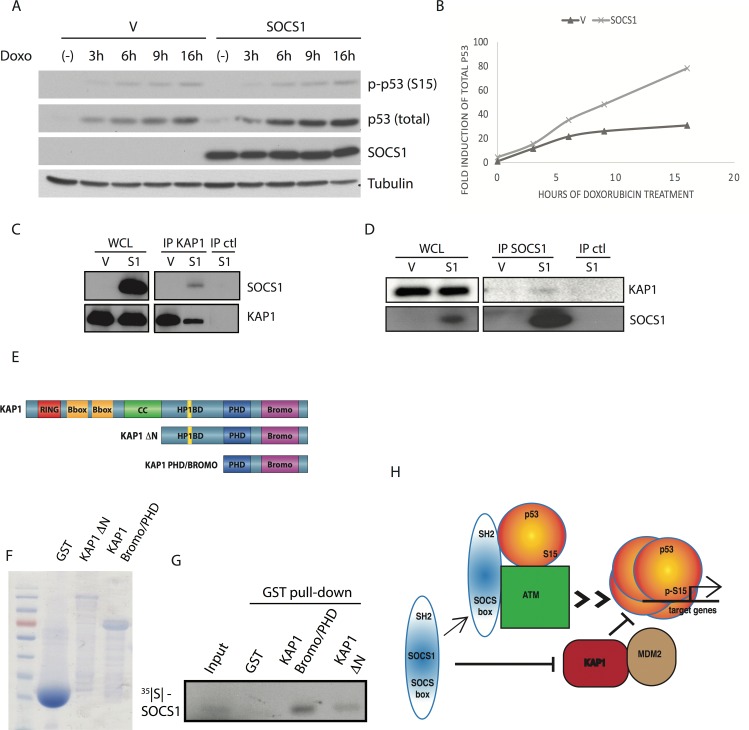
SOCS1 favors p53 accumulation in response to Doxorubicin (**A**) Western blots of SOCS1, phosphorylated p53 at serine 15 [p-p53 (S15)] total p53 and tubulin in IMR90 cells expressing either empty vector (V) or SOCS1 and treated with doxorubicin (Doxo: 300 ng/mL) for 3, 6, 9, 16 hours or untreated (−). (**B**) Graphic representation of Western blots as in (A). Bands were quantified using image analysis software and normalized to tubulin, then plotted in a graph to show the kinetics of p53 stabilization. (**C**) Co-Immunoprecipitation of KAP1 with SOCS1. U2OS cell lysates of either empty vector cells (V) or SOCS1 overexpressing cells (S1) were immunoprecipitated with an antibody against KAP1 or a control antibody (IP ctl). Western blots against both KAP1 and SOCS1 were performed to confirm the presence of SOCS1 in complex with KAP1. Whole cell lysates (WCL) are used to control the expression of SOCS1 and KAP1 levels. (**D**) Co-immunoprecipitation as described in C. Cell lysates were immunoprecipitated with an antibody against SOCS1 or with a control antibody (IP ctl). Whole cell lysates (WCL) show the expression level of SOCS1 and KAP1. (**E**) Maps of the different KAP1 constructs used in experiments are depicted: KAP1 full length (KAP1), KAP1 with a deletion of its N-terminal RBCC domains (KAP1 ΔN) or KAP1 C-terminus including PHD and Bromo domains (KAP1 PHD/BROMO). (**F**) The constructs depicted in E. were expressed by IPTG induction in BL21 bacterial cells. Expression levels of the various constructs were assessed by migration of an SDS-PAGE gel and Coomassie staining. (**G**) GST pull down was performed on KAP1 constructs which were incubated with radiolabeled SOCS1. Autoradiography revealed the absence or presence of SOCS1 in each pull down. GST was used as a negative control. (**H**) Model for p53 activation by SOCS1 via two pathways.

To investigate how SOCS1 modulates p53 activity, we first induced endogenous p53 using the DNA damaging drug doxorubicin and measured both p53 phosphorylation and total p53 levels. SOCS1 expression increased p53 levels during the course of a doxorubicin treatment without changing the levels of serine 15-phosphorylation of p53, which are highly induced by the drug (Figure 7A and B). In other words, in the context of oncogenic stress that involves relatively modest rates of DNA damage, SOCS1 stimulates serine 15 phosphorylation of p53 by promoting p53-ATM interactions as described before [14]. In cells treated with doxorubicin, high levels of DNA damage maximally stimulate serine 15 phosphorylation of p53 in a SOCS1-independent manner but SOCS1 can still stabilize p53 in this condition.

In order to find additional mechanisms of p53 activation by SOCS1, we looked at the SOCS1 interactome [15]. Interestingly, KAP1, a repressor of p53 [39], immunoprecipitated with SOCS1 [15]. To confirm this finding we expressed wild type SOCS1 in U2OS cells and immunoprecipitated endogenous KAP1 using a specific antibody. We found SOCS1 in KAP1 immunoprecipitates but not in immunoprecipitates obtained with a control antibody (Figure 7C). The reciprocal co-IP analysis confirmed that endogenous KAP1 interacted with SOCS1 (Figure 7D). Next, we used a GST-pull-down assays with two fragments of KAP1 and an *in vitro* translated and ^35^S labelled SOCS1. We found that the Bromo/PHD domain of KAP1 directly interacted with SOCS1 (Figure 7E-G). We thus propose that SOCS1 activates the senescence functions of p53 via two mechanisms (Figure 7H): 1) facilitating serine 15 phosphorylation of p53 and 2) p53 stabilization by interfering with KAP1. Together, these mechanisms can contribute to convert an acute p53 response into a chronic response characterized by the expression of a unique pattern of p53 target genes.

## DISCUSSION

The tumor suppressor activity of the transcription factor p53 does not correlate with the expression of target genes induced by acute DNA damage [6]. The discovery and characterization of p53 targets in conditions of chronic stimulation of the p53 pathway should give critical insights into the mechanisms of tumor suppression by p53. Oncogene-induced senescence is a tumor suppressor mechanism where a lasting p53 response mediates a stable cell cycle arrest and the clearance of senescent cells, which prevents tumor progression [40]. The suppressor of cytokine signaling SOCS1 was previously shown to be required for p53 activation and senescence in response to constitutive JAK-STAT5 signaling [14,15,41,42]. However, the p53 target genes requiring SOCS1 remained poorly characterized. Here, we used RNA interference and transcriptome analysis to identify the set of SOCS1-dependent p53 target genes. Although SOCS1 was required for the expression of several previously identified p53 targets, most of the classic targets associated with acute DNA damage response such as p21 and MDM2 were not affected by SOCS1 inhibition (Table 1). In addition, bioinformatics analysis using the platform DiRE, uncovered several genes that required SOCS1 expression and contained p53-binding sites in their promoter regions (Table 2). This unbiased transcriptome analysis confirms that SOCS1 regulates the p53 pathway and reveals a unique and interesting biology for the SOCS1-p53 axis.

The identification of SOCS1-dependent p53 targets links p53 to several interesting tumor suppression pathways. For example, DDIT-3 (also known as C/EBP Zeta) is a dominant negative inhibitor of C/EBP family of transcription factors [43]. This family is important for the expression of inflammatory cytokines in senescent cells that contribute to the SASP [44,45]. SOCS1 may regulate the SASP via p53 and DDIT-3 (Table 2). In addition, several SOCS1-dependent p53 targets are secreted proteins suggesting that the SOCS1-p53 pathway changes the quality of the SASP. They include the bona fide p53 targets IGFBP3, IGFBP7, SERPINE1/PAI1, DKK1 and GDF15 (Table 1) and the new candidate p53 targets ESM1 and SRPX2 (Table 2). Another interesting target is GADD45B that mediates activation of the p38MAPK pathway, which is required for OIS [46]. Perhaps the most intriguing connection found by our analysis involves the ferroptosis pathway. This form of cell death was linked to p53-dependent tumor suppression and involves the SOCS1-dependent p53 targets SLC7A11 [4] and SAT1 [24]. In addition, ferroptosis is mediated by oxidized lipids [23] and the overall gene expression pattern of SOCS1-expressing cells overlaps with genes regulated by oxidized lipids (Figure S3D). The SOCS1-dependent p53 targets DDIT3, PMAIP1, ATF3 and ESM1 are part of the gene set induced by oxidized phospholipids and therefore bona fide new candidates to regulate ferroptosis downstream the SOCS1-p53 axis.

SOCS1 does not only play a role in the senescence response to constitutively active JAK-STAT5 signaling. In cells expressing oncogenic RAS, SOCS1 is recruited to DNA damage foci, colocalizing with phospho-ATM. The extent to which SOCS1 modifies gene expression in RAS-induced senescence remains to be fully characterized. However, DKK1, GDF15, Noxa, Dusp1 and SLC7A11 were modulated in Ras- or PML-expressing cells in the same way as in cells expressing STAT5A or SOCS1. In contrast to SOCS1-cells, RAS- or STAT5A-senescent cells were not more sensitive to ferroptosis induced by TBH, suggesting that other pathways activated in these cells control ferroptosis. Intriguingly, PML expressing cells were highly resistant to ferroptosis providing a mechanistic insight into ferroptosis resistance pathways. In addition to influencing p53 target gene expression, SOCS1 could play a general role in senescence by stabilizing the interactions of p53 with protein complexes at DNA damage foci. This would allow the maintenance of a pool of pre-active p53 that can be slowly released during the senescence cell cycle arrest contributing to generate a lasting chronic p53 response. Another function for maintaining p53 in DNA damage foci could be to suppress homologous recombination in cells arrested in G1 [47,48], an event that could lead to chromosome aberrations and potentially tumor development.

In summary, we report here that SOCS1 impacts the pattern of secreted products in cells with active p53 and is required for the expression of a selective set of p53 target genes including those involved in ferroptosis. SOCS1 can use several mechanisms to activate p53, including promotion of serine-15 phosphorylation by ATM/ATR kinases and inhibition of the p53 repressor KAP1. Further investigation of the SOCS1-p53 pathway will help to better understand p53 tumor suppression activity and provide insights for novel cancer therapies.

## MATERIALS AND METHODS

### Cell lines, reagents, growth analysis and senescence

U2OS were purchased from the American Type Culture Collection, (Manassas, VA) and normal human diploid fibroblasts IMR90 were purchased from the Coriell Institute (Camden, New Jersey, USA). IMR90 were cultured in DMEM supplemented with 10% fetal bovine serum (FBS; Wisent, Montréal, QC, Canada) and 1% penicillin G/streptomycin sulphate. U2OS were supplemented with 5% FBS (Wisent) and 5% Newborn Calf serum (Wisent), with 1% penicillin G/streptomycin sulphate and with 2mM L-glutamine. Tert-butyl Hydroperoxide was purchased from Sigma (cat #458139) and used at 350 μM in U2OS cells and 88 μM in IMR90 cells. Doxorubicin was purchased from Sigma. Growth curves and the senescence associated β-galactosidase were performed as previously described [49].

### Plasmid constructions and viral gene transfer

Ca-STAT5A was previously described in [50]. pLPC-SOCS1 was previously described in [14]. pLPC PML IV and pBabe RASV12 were previously described in [49]. GST-KAP1 constructs were a kind gift of Dr. Xavier Mascle. Lentiviral shRNAs against SOCS1 were purchased from Sigma-Aldrich in the pLKO vector. ShSOCS1a (shS1) (TRCN0000356245, Sigma) has the following sequence: CCGGCTGGTTGTTGTAGCAGCTTAACTCGAGTTAAGCTGCTACAACAACCAGTTTTTG and shSOCS1b (TRCN0000057065, Sigma) has the following sequence: CCGGCTTCCGCACATTCCGTTCGCACTCGAGTGCGAACGGAATGTGCGGAAGTTTTTG. shNTC has the following sequence: CCGGCAACAAGATGAAGAGCACCAACTCGAG TTGGTGCTCTTCATCTTGTTGTTTTT. pLXSN E7 was a kind gift from Dr. D. Galloway. Retroviral gene transduction was performed as previously described [49]. Lentiviral gene transduction was performed by co-transfecting 6 μg of the lentiviral vector with the packaging vectors in 293T cells as follows: 3 μg of the VSV-G envelope protein expression plasmid pMD2, 1.5 μg of the regulator of virion expression (REV) expression plasmid pRSV and 1.5 μg of gag/pol elements expression plasmid pMDLg/pRRE. 24 hours post transfection, media were changed and the supernatants (viral soup) were collected 48 hours post transfection, filtered through 0.45 μm filters (Sarstedt) and added to target cells together with DMEM and 4 μg/mL polybrene. Viruses were removed after 8 hours and replaced by fresh medium. Selection was started 24 hours post-infection. For triple infections in IMR90 cells, G418 (300 μg/mL, 6 days) and puromycin (1.5 μg/mL, 3 days) were used first. After puromycin selection was over, hygromycin (80 μg/mL) was added for 4 days. Cells were selected for a total of 7 days before RNA collection. For U2OS and IMR90 single infections, puromycin was used at 3 μg/mL for 3 days.

### Ferroptosis cell death assays

U2OS and IMR90 cells were seeded 24 hours prior to treatments in 10 cm plates (Corning) at 60% confluence. Cells were then treated with 350 μM (U2OS) or 88 μM (IMR90) TBH (Sigma #458139) for 16 hours. Cell death was assessed by imaging the cells under a white-light microscope and by counting live and dead cells using Trypan-Blue (BioRad) and a cell counter. Supernatants were collected and added to trypsinized cells before counting. All assays were performed at least three times. For IC50 assays, 20 000 IMR90 cells of each condition were plated per well of a 48 well plate. Cells were plated 24 hours prior to TBH treatment. Cells were then treated with either: 0, 10, 20, 40, 60, 80, 90, 100, 120, 160, 180 or 200 μM of TBH for 16 hours. Cells were then fixed with 1% gluteraldehyde in PBS and stained with Crystal Violet (Sigma #C0775). The dye was then resuspended in 10% acetic acid and dosed with a spectrophotometer. GraphPad Prism was used to generate IC50 curves and determine IC50 values.

### Western blotting

Protein analysis was performed by lysing cells in Cell Lysis Buffer (20 mM Tris-HCl pH 7.5, 150 mM NaCl, 1 mM EDTA, 1 mM EGTA, 1% Triton X-100, 2.5 mM sodium pyrophosphate, 1 mM β-glycerolphosphate and a cocktail of protease inhibitors (Roche). Quantification of protein content was performed with the Bradford method. Extracts were prepared with Laemmli buffer (1X final). For endogenous SOCS1 detection, 150 μg of total extract was loaded on a 12% SDS-PAGE gel and transferred on an Immobilon P membrane (Millipore). For SOCS1 overexpression and other protein detection, 50 μg of total cells extract was loaded on a 12% SDS polyacrylamide gel. Western blots were performed as described previously [14]. The following primary antibodies were used: anti-SOCS1, clone 4H1 MBL (cat #K0175-6) used at 1:1000 dilution overnight at 4°C. Anti-phospho-p53 (Serine15), NEB (cat #9284) used at 1:1000 dilution overnight at 4°C. Total p53 (DO1 clone), Santa Cruz biotechnology (cat# sc-126) used at 1:1000 overnight at 4°C. Anti-MCM6, Bethyl (#A300-194A) used at 1:1000 overnight at 4°C. Anti-phosphorylated-Rb (Serine 795), Cell Signaling (#9301) used at 1:1000 overnight at 4°C. Anti-phosphorylated Histone H3 (Serine 10), Millipore (#6570) used at 1:1000 overnight at 4°C. Alpha Tubulin antibody clone B-5-1-2, Sigma (cat # T6074) used at 1:20,000 1 hour at Room Temperature and served as loading control. Signals were revealed by using secondary antibodies coupled to peroxidase (BioRad laboratories) and ECL (GE Healthcare, cat# RPN2106) or Clarity ECL (BioRad #cat 1705061).

### Co-immunoprecipitation

U2OS cells were collected in Cell Lysis Buffer (20 mM Tris-HCl pH 7.5, 150 mM NaCl, 1 mM EDTA, 1 mM EGTA, 1% Triton X-100, 2.5 mM sodium pyrophosphate, 1 mM β-glycerolphosphate and a cocktail of protease inhibitors (Roche)) and protein concentration measured using the Bradford method. 2 mg of cell extract from each condition was used for IP and 50 ug was loaded as input (Whole Cell Lysate). Immunoprecipitation of either KAP1 (Bethyl #A300-274A) or SOCS1 (SantaCruz #sc-7005R) were performed at an antibody dilution of 1:200 overnight at 4°C. Protein A Sepharose 4B fast flow beads (Sigma #P9424) were used to immunoprecipitate antibody-protein complexes for 1 hour at 4°C. Beads were then washed four times with Cell Lysis Buffer and complexes were eluted by adding Laemmli 2X buffer directly to the beads. Samples were heated for 5 minutes at 100°C prior to loading on an SDS-PAGE gel for western blotting.

### GST-pull-down

BL21 *E. coli* strain harboring each of the KAP1-GST-fusion vectors were grown at 37°C to an OD of 0.6 in 400 ml YTA 2X medium (16g/l tryptone, 10g/l yeast extract, 5g/l NaCl) and induced with 0.4 mM IPTG for 4 hr at 30°C. Cell pellets were resuspended in STE buffer (10 mM Tris-HCl pH 8.0, 1 mM EDTA, 100 mM NaCl) supplemented with DTT (5mM) and a Protease Inhibitor Cocktail (Roche). Cells were lysed by adding 1mg/mL lysozyme and incubating 45 minutes on ice prior to sonicating with a microtip five times for 10 s. Extracts were centrifuged (13 000 rpm 10 minutes) and supernatants were incubated for 2 hr at 4°C with glutathione Sepharose-4B (Amersham) and washed four times with NETN buffer (10 mM Tris-HCl pH 8.0, 1 mM EDTA, 100 mM NaCl, 0.5% NP-40). Beads were resuspended in PBS after the last wash.

Briefly, ^35^[S] Flag-SOCS1 was produced by *in vitro* transcription/translation (Promega #L1170) and incubated with GST, GST-KAP1, GST KAP1 PHD/BROMO or GST-KAP1 ΔN purified proteins bound to glutathione Sepharose-4B for 2 hr at 4°C. Equal amounts of GST fusion proteins were used as judged from SDS gel electrophoresis and Coomassie blue staining. Beads were washed 5 times with NETN buffer. Precipitates were eluted in 20 μl of SDS Sample Buffer and 10 μl of Bromophenol Blue and boiled for 5 min. Eluates (30 μl) were separated on SDS-PAGE, dried and exposed for autoradiography.

### GSH quantification

Cells were collected in Cell Lysis Buffer (20 mM Tris-HCl pH 7.5, 150 mM NaCl, 1 mM EDTA, 1 mM EGTA, 1% Triton X-100, 2.5 mM sodium pyrophosphate, 1 mM β-glycerolphosphate and a cocktail of protease inhibitors (Roche)). GSH was then quantified with the QuantiChrom Glutathione (GSH) Assay Kit (BioAssay Systems, DIGT-250) following manufacturer's instructions. Quantification was normalized to total protein concentration of each sample.

### Real-Time Quantitative PCR (qPCR)

Total RNA was isolated using Trizol (Invitrogen). Reverse transcription was performed using 5X All-In-One RT MasterMix (Abmgood) on 2 μg of total RNA in 20 μL final volume according to the kit's instructions. Reverse transcription products were diluted 10-fold in RNAse free water before proceeding to qPCR. QPCR was performed using primers and probe sets from Roche Universal Probe Library (https://lifescience.roche.com/en_ca/brands/universal-probe-library.html?_ga=1.38905443.192324701.1470126343#overview). 96-well plate formats with SYBR-green technology was used as described previously [26]. Relative target-gene quantification was obtained by using the ΔΔCT method in a light Cycler 480 (Roche). The mRNA expressions were measured relative to the mRNAs of two housekeeping genes: HMBS and TBP. All forward and reverse primers are listed in Table 4.

**Table 4:** 

Gene symbol	Forward primer	Reverse primer
DUSP1	caacgaggccattgacttcataga	atggtggctgaccgggaaat
IGFBP3	tctcccaggctacaccaccaa	ggcatatttgagctccacattaacct
IGFBP5	ccgcgagcaagtcaagatcg	taggtctcctcggccatctca
ATF3	tgaggtttgccatccagaacaa	tttcatcttcttcaggggctacc
SLC7A11	ctccatgaacggtggtgtgttt	ccctctcgagacgcaacataga
ALDH1L2	tctggctttggaaaagacttagg	cctgatgatggtgttgctctaat
Serpine1	cggtcaagcaagtggacttttc	ggctcctttcccaagcaagtt
NOXA	gaagaaggcgcgcaagaacg	tgagtagcacactcgacttcca
RGCC	cgccacttccactacgaggag	cactgaagctgaagctgttcct
IGFBP7	cctgtcctcatctggaacaaggt	tctgaatggccaggttgtcc
DKK1	atgatcatagcaccttggatggg	gcacaacacaatcctgaggcaca
DDIT3	catacatcaccacacctgaaagca	gctggtctgatgcctgtttttgt
PPP1R3C	agcgggtgctggcttttagg	tggatctaaaacctggatcattctg
NOLC1	gcggcagtggtagtttccaaat	tgaagctttatcttcttggcctga
NTN4	cgtgcacaataagagcgaacca	tgttccttacattcgcatttacctg
SVEP1	tctgttggtttgcccatacctg	ttatggagcccacaaaagactc
PROCR	aacattgctgccgatactgctg	tctggagcatatgaagtctttgga
ESM1	catggatggcatgaagtgtg	ccagatgccatgtcatgctcttt
IGFBP4	gcaacttccaccccaagcag	cggtccacacaccagcactt
IGFBP6	aggaatccaggcacctctacca	agtccagatgtctacggcatgg
SOCS1	ggtccccctggttgttgta	taggaggtgcgagttcaggt
GDF15	agtccggatactcacgccagaa	gcccgagagatacgcaggtg
GADD45B	tgcattgtctcctggtcacgaa	cccggctttcttcgcagtag

### Microarray analysis

RNA was collected from IMR90 cells expressing oncoprotein E7, seven days after co-infection with pWZL ca-STAT5A (5A) and pLKO shNTC or pWZL 5A and pLKO shSOCS1 a. Total RNA samples were isolated with the RNeasy mini kit (Qiagen) and sent to the Genome Quebec facility at McGill University for cRNA amplification and subsequent hybridization on GeneChIP Human Gene 2.0 ST Array Affymetrix DNA Chip. Data were analyzed using Affymetrix Expression Console Software and Transcriptome Analysis Console (www.affymetrix.com). Each condition was analysed in biological triplicates and the cut-off applied for analysis was an ANOVA p-value <0.05 for genes that have a fold change ≥±1.5. Data are available at https://www.ncbi.nlm.nih.gov/geo/query/acc.cgi?acc=GSE98216 Further analysis were conducted with DiRE (https://dire.dcode.org/), Gene-Set Enrichment Analysis (GSEA) (http://software.broadinstitute.org/gsea/index.jsp) and DAVID database (https://david.ncifcrf.gov/). Genes identified by DiRE were further analysed with Oncomine (https://www.oncomine.org/resource/login.html).

### Gene expression analysis in hepatocellular carcinoma (HCC) specimens

The correlation between *SOCS1* gene expression and that of p53 target genes related to ferroptosis was analysed using The Cancer Genome Atlas (TCGA) [51] provisional dataset containing 373 hepatocellular carcinoma (HCC) specimens, which was accessed via the cBioportal (http://www.cbioportal.org) [52]. The transcript levels were expressed as RNASeq Ve RESM (RNA-Seq by Expectation Maximization) [53]. The downloaded data were plotted using the GraphPad Prism software to determine the Spearman correlation (ρ) and statistical significance (*p*).

## SUPPLEMENTARY MATERIAL FIGURES


