# Supplementary material for: SOCS1 regulates senescence and ferroptosis by modulating the expression of p53 target genes
Source: Aging (Albany NY). 2017 Oct 28;9(10):2137–62. doi: 10.18632/aging.101306 (PMC5680560; doi:10.18632/aging.101306)
Supplement: Supplementary file 1 [file aging-09-2137-s001.pdf]

Research Paper

SUPPLEMENTAL MATERIAL

A

Category	Term	Genes	Count	%	P-Value	Benjamini
SP_PIR_KEY WORDS	Secreted		87	19.7	2.90E-15	1.10E-12
SP_PIR_KEY WORDS	signal		119	26.9	1.70E-10	3.30E-08
SP_PIR_KEY WORDS	glycoprotein		137	31	5.30E-08	7.00E-06
SP_PIR_KEY WORDS	disulfide bond		100	22.6	2.90E-07	2.80E-05
SP_PIR_KEY WORDS	egf-like domain		19	4.3	1.60E-06	1.20E-04
SP_PIR_KEY WORDS	extracellular matrix		18	4.1	1.20E-05	8.10E-04
SP_PIR_KEY WORDS	Growth factor binding		5	1.1	2.70E-04	1.50E-02
SP_PIR_KEY WORDS	cell adhesion		20	4.5	1.40E-03	6.60E-02

B

Genes from "Secreted" group	GENE NAME	Related Genes	Species
Kazal-type serine peptidase inhibitor domain 1	Kazal-type serine peptidase inhibitor domain 1	RG	Homo sapiens
connective tissue growth factor	connective tissue growth factor	RG	Homo sapiens
endothelial cell-specific molecule 1	endothelial cell-specific molecule 1	RG	Homo sapiens
insulin-like growth factor binding protein 3	insulin-like growth factor binding protein 3	RG	Homo sapiens
insulin-like growth factor binding protein 4	insulin-like growth factor binding protein 4	RG	Homo sapiens
insulin-like growth factor binding protein 5	insulin-like growth factor binding protein 5	RG	Homo sapiens
insulin-like growth factor binding protein 6	insulin-like growth factor binding protein 6	RG	Homo sapiens
insulin-like growth factor binding protein 7	insulin-like growth factor binding protein 7	RG	Homo sapiens

C

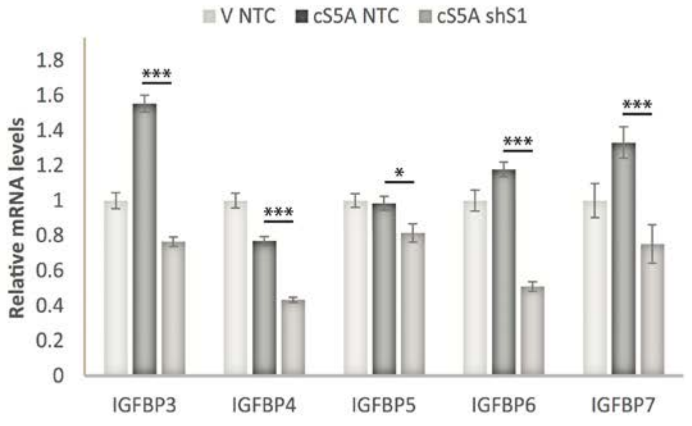

Supplementary Figure S1. David Analysis identifies Secreted proteins as differentially regulated in cells with SOCS1 knockdown. (**A**) DAVID Analysis of Key words category. (**B**) Genes differentially regulated in the "Secreted" category as identified by David Analysis. (**C**) QPCR validation of secreted factors of the IGFBP family identified by David Analysis in IMR90 cells expressing E7 in combination with either an empty vector (V) or with constitutively activated STAT5A (cS5A) and with either a control shRNA (shNTC) or an shRNA against SOCS1 (shS1). All experiments were performed three times, error bars indicate standard errors of triplicates *= p<0.05, using the Student's t test, **=p<0.01, ***=p<0.005.

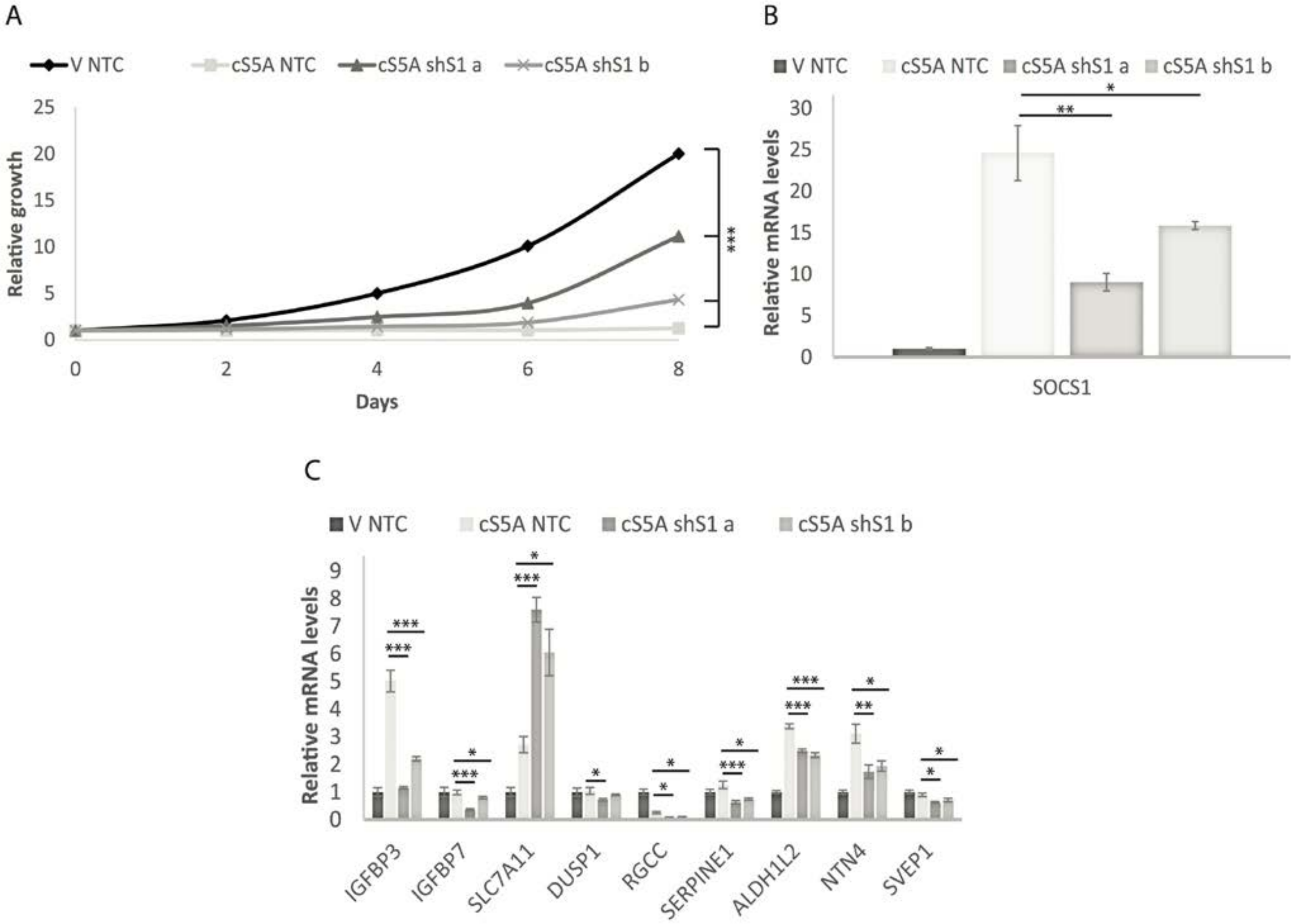

Supplementary Figure S2. Validation of SOCS1-dependent p53 target genes with two shRNAs against SOCS1. (**A**) Growth curves of IMR90 cells expressing E7 with either an empty vector (V) or with constitutively activated STAT5A (cS5A) and with either a control shRNA (NTC) or one of two shRNAs (shS1a or shS1b). (**B**) SOCS1 mRNA levels were measured by qPCR in IMR90 cells expressing the same constructs as described in (A) to assess SOCS1 knockdown efficiency. (**C**) QPCR of SOCS1-dependent p53 target genes in the conditions described previously. All experiments were performed three times, error bars indicate SD of triplicates (growth curves) or standard errors of triplicates (QPCR), *= p<0.05, using the Student's t test, **=p<0.01 and ***=p<0.005.

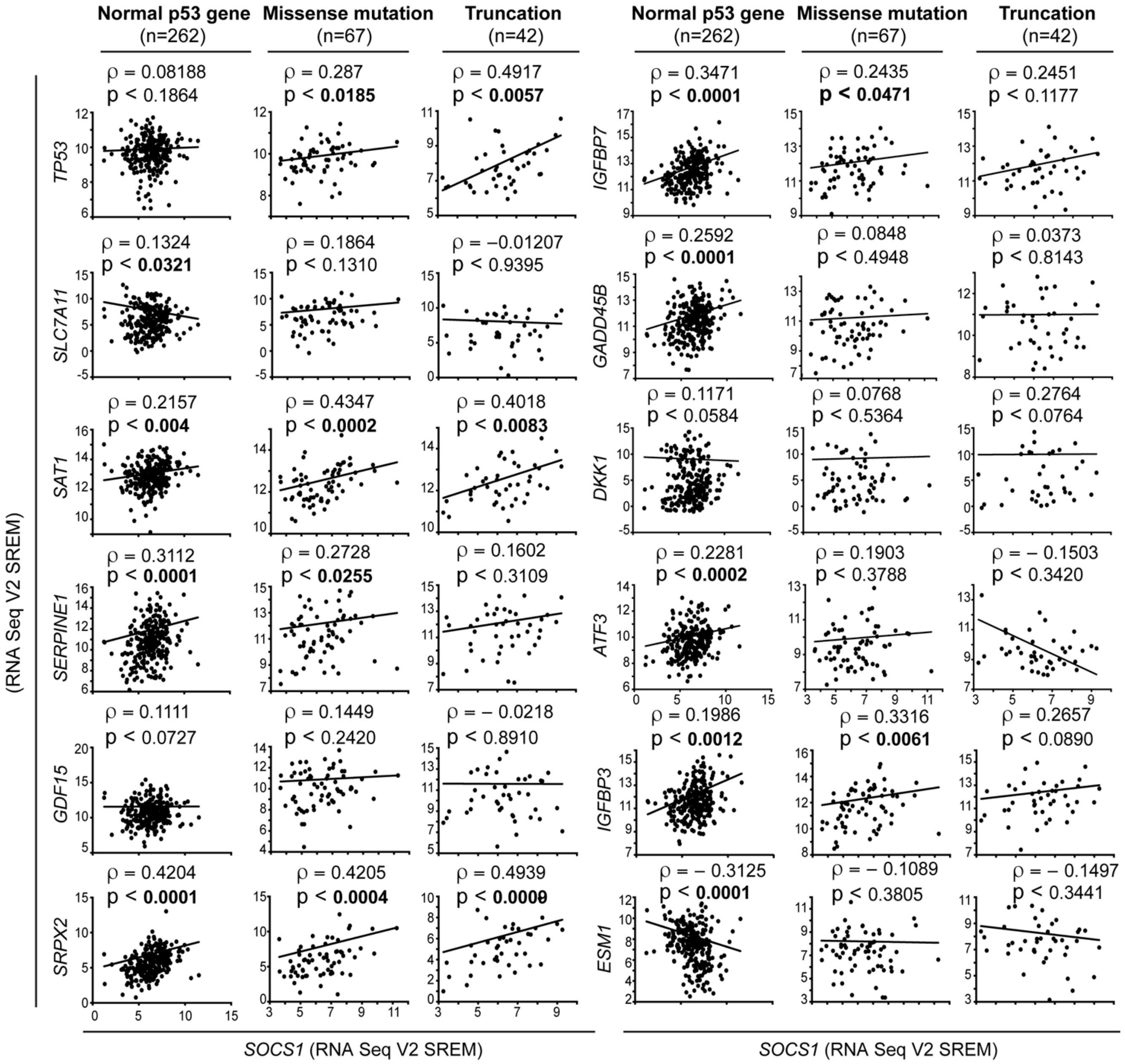

Supplementary Figure S3. Correlation between SOCS1 and p53-target gene expression in hepatocellular carcinoma samples separated according to p53 status. The Spearman correlation (ρ) and the *p* values are given at the top of each plot.

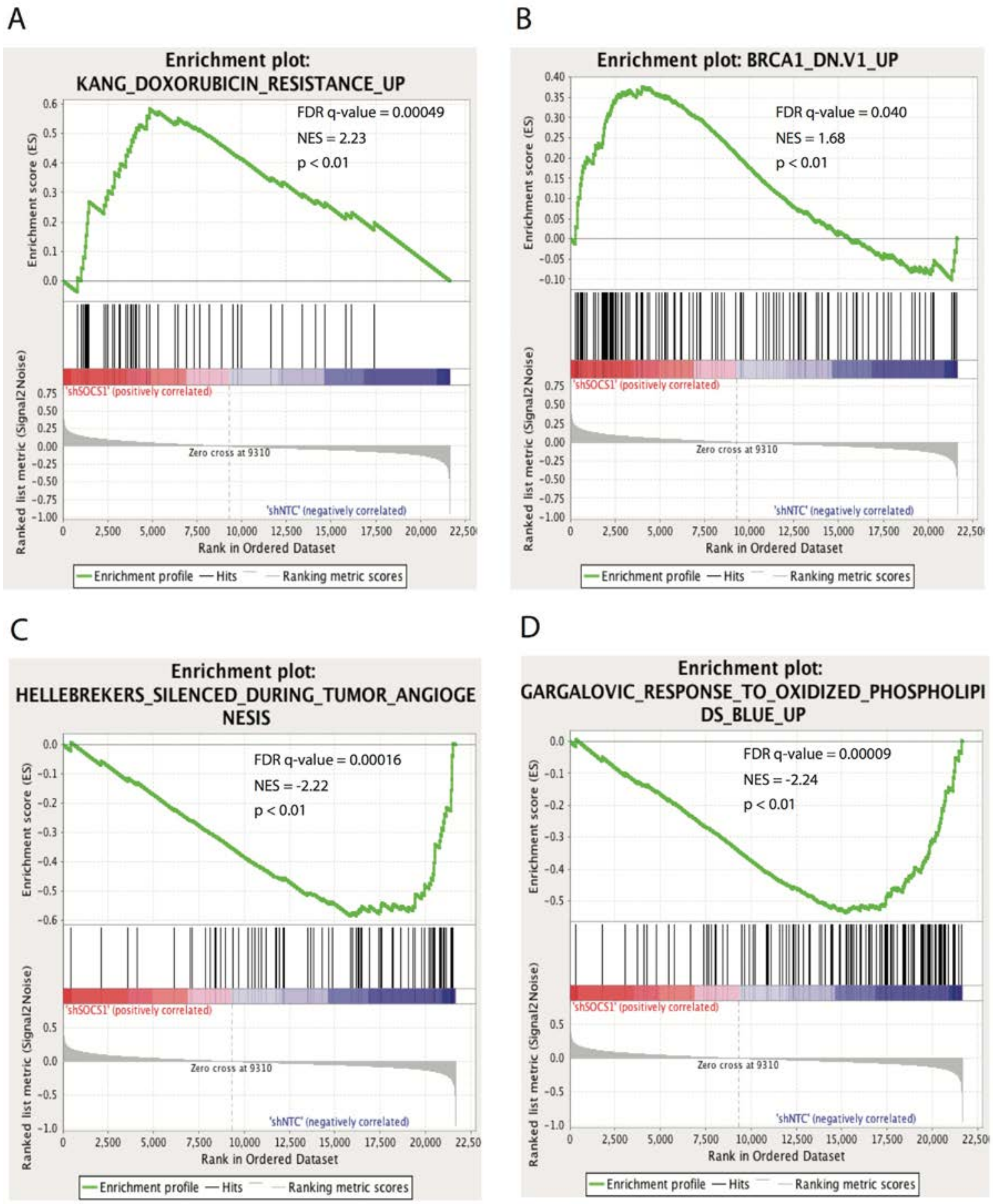

Supplementary Figure S4. GSEA analysis identifies genes sets associated with p53, angiogenesis and lipid oxidation as differentially regulated by SOCS1 knockdown. (**A**) Genes upregulated in a doxorubicin resistance context correlate with genes upregulated in presence of shSOCS1. (**B**) Genes upregulated in a BRCA1 dominant negative context are upregulated in presence of shSOCS1. (**C**) Genes silenced during tumor angiogenesis correlate with genes upregulated in the presence of SOCS1. (**D**) Genes upregulated in response to oxidized phospholipids are enriched in the presence of SOCS1.

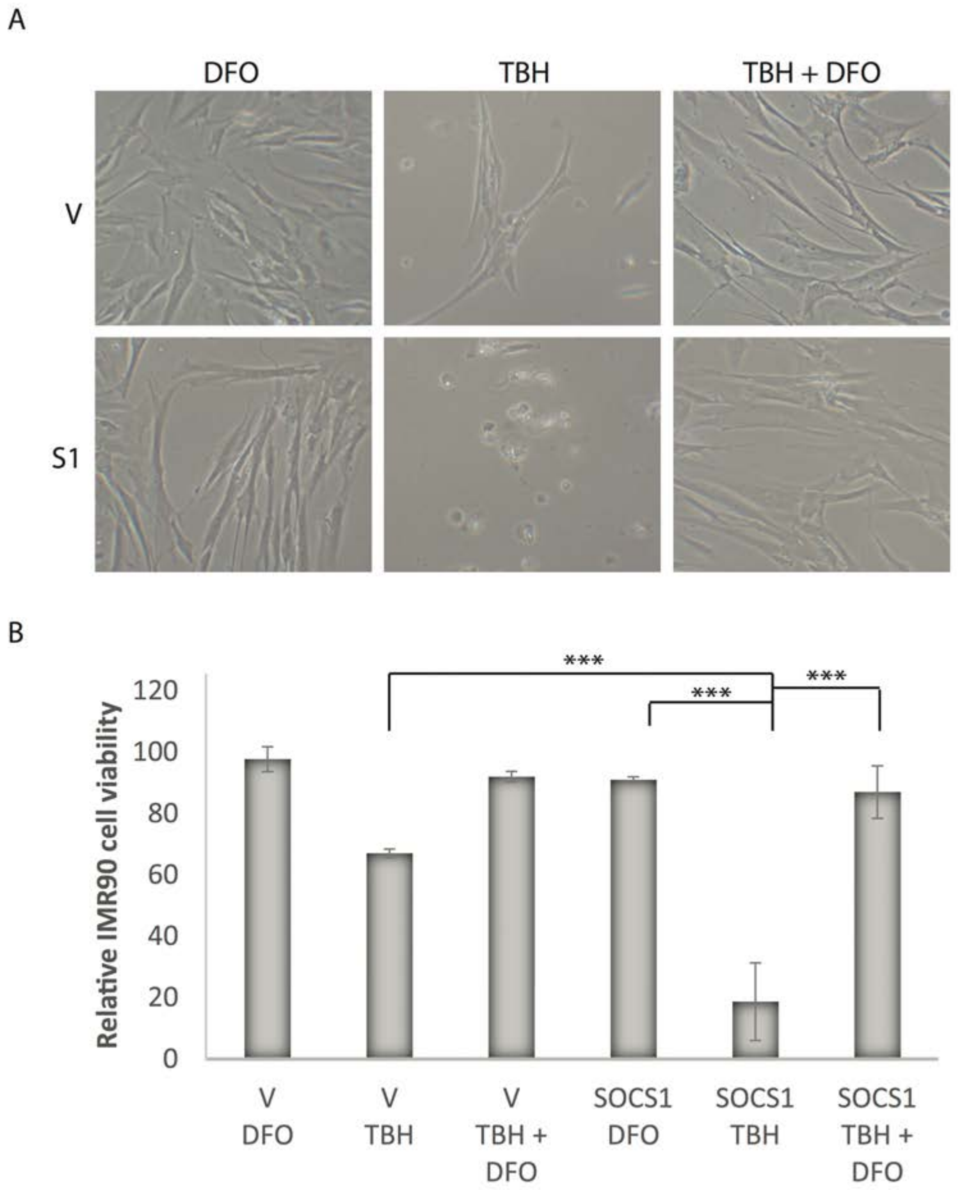

Supplementary Figure S5. DFO rescues TBH-induced ferroptosis in IMR90 cells. (**A**) Representative photos of IMR90 cells expressing either V or S1 by retroviral infection and treated 24 hours after plating with either 88 µM tert-butyl-hydroperoxide (TBH) alone, 100 µM Deferoxamine mesylate (DFO) alone or the combination of both drugs. (**B**) Quantification of cell viability of cells portrayed in A. All experiments were performed three times, error bars indicate SD of triplicates, *= p<0.05, using the Student's t test, **=p<0.01, ***=p<0.005.
